# Climate-change-driven growth decline of European beech forests

**DOI:** 10.1038/s42003-022-03107-3

**Published:** 2022-03-10

**Authors:** Edurne Martinez del Castillo, Christian S. Zang, Allan Buras, Andrew Hacket-Pain, Jan Esper, Roberto Serrano-Notivoli, Claudia Hartl, Robert Weigel, Stefan Klesse, Victor Resco de Dios, Tobias Scharnweber, Isabel Dorado-Liñán, Marieke van der Maaten-Theunissen, Ernst van der Maaten, Alistair Jump, Sjepan Mikac, Bat-Enerel Banzragch, Wolfgang Beck, Liam Cavin, Hugues Claessens, Vojtěch Čada, Katarina Čufar, Choimaa Dulamsuren, Jozica Gričar, Eustaquio Gil-Pelegrín, Pavel Janda, Marko Kazimirovic, Juergen Kreyling, Nicolas Latte, Christoph Leuschner, Luis Alberto Longares, Annette Menzel, Maks Merela, Renzo Motta, Lena Muffler, Paola Nola, Any Mary Petritan, Ion Catalin Petritan, Peter Prislan, Álvaro Rubio-Cuadrado, Miloš Rydval, Branko Stajić, Miroslav Svoboda, Elvin Toromani, Volodymyr Trotsiuk, Martin Wilmking, Tzvetan Zlatanov, Martin de Luis

**Affiliations:** 1 Department of Geography, Johannes Gutenberg University, Mainz, Germany; 2 Department of Forestry, University of Applied Sciences Weihenstephan-Triesdorf, Triesdorf, Germany; 3 Land Surface-Atmosphere Interactions, Technical University Munich, Freising, Germany; 4 Department of Geography and Planning, School of Environmental Sciences, University of Liverpool, Liverpool, UK; 5 Global Change Research Institute of the Czech Academy of Sciences (CzechGlobe), Brno, Czech Republic; 6 Department of Geography, Autonomous University of Madrid, Madrid, Spain; 7 Nature Rings – Environmental Research and Education, Mainz, Germany; 8 Plant Ecology, Albrecht-von-Haller-Institute for Plant Sciences, University of Goettingen, Goettingen, Germany; 9 Forest Dynamics, Swiss Federal Research Institute for Forest, Snow and Landscape WSL, Birmendorf, Switzerland; 10 School of Life Science and Engineering, Southwest University of Science and Technology, Mianyang, China; 11 Department of Crop and Forest Sciences and Joint Research Unit CTFC-AGROTECNIO CERCA Center, University of Lleida, Lleida, Spain; 12 Institute for Botany and Landscape Ecology, University Greifswald, Greifswald, Germany; 13 Systems and Natural Resources Department, Universidad Politécnica de Madrid, Madrid, Spain; 14 Chair of Forest Growth and Woody Biomass Production, TU Dresden, Tharandt, Germany; 15 Biological and Environmental Sciences, Faculty of Natural Sciences, University of Stirling, Stirling, Scotland; 16 Faculty of Forestry and Wood Technology, University of Zagreb, Zagreb, Croatia; 17 Institute of Forest Ecosystems, Thünen Institute, Eberswalde, Germany; 18 TERRA Teaching and Research Centre (Forest Is Life), Gembloux Agro-Bio Tech, University of Liege, Gembloux, Belgium; 19 Faculty of Forestry and Wood Sciences, Czech University of Life Sciences, Prague, Czech Republic; 20 Biotechnical Faculty, Department of Wood Science and Technology, University of Ljubljana, Ljubljana, Slovenia; 21 Applied Vegetation Ecology, Faculty of Environment and Natural Resources, University of Freiburg, Freiburg, Germany; 22 Slovenian Forestry Institute, Ljubljana, Slovenia; 23 Forest Resources Department, Centro de Investigación y Tecnología Agroalimentaria de Aragón (CITA), Zaragoza, Spain; 24 University of Belgrade – Faculty of Forestry, Belgrade, Serbia; 25 Department of Geography and Regional Planning, University of Zaragoza, Zaragoza, Spain; 26 TUM School of Life Sciences/Ecoclimatology, Technical University of Munich, Munich, Germany; 27 Department of Agriculture, Forestry and Food Sciences, University of Turin, Grugliasco, Italy; 28 Department of Earth and Environmental Sciences, University of Pavia, Pavia, Italy; 29 National Institute for Research and Development in Forestry “Marin Dracea”, Voluntari, Romania; 30 Faculty of Silviculture and Forest Engineering, University of Brasov, Brașov, Romania; 31 Departamento de Sistemas y Recursos Naturales, Escuela Técnica Superior de Ingeniería de Montes, Forestal y del Medio Natural, Universidad Politécnica de Madrid, Madrid, Spain; 32 Faculty of Forestry Sciences, Agricultural University of Tirana, Koder-Kamez, Albania; 33 Institute of Biodiversity and Ecosystem Research, Bulgarian Academy of Sciences, Sofia, Bulgaria

**Keywords:** Forest ecology, Drought, Climate-change ecology

## Abstract

The growth of past, present, and future forests was, is and will be affected by climate variability. This multifaceted relationship has been assessed in several regional studies, but spatially resolved, large-scale analyses are largely missing so far. Here we estimate recent changes in growth of 5800 beech trees (*Fagus sylvatica* L.) from 324 sites, representing the full geographic and climatic range of species. Future growth trends were predicted considering state-of-the-art climate scenarios. The validated models indicate growth declines across large region of the distribution in recent decades, and project severe future growth declines ranging from −20% to more than −50% by 2090, depending on the region and climate change scenario (i.e. CMIP6 SSP1-2.6 and SSP5-8.5). Forecasted forest productivity losses are most striking towards the southern distribution limit of *Fagus sylvatica*, in regions where persisting atmospheric high-pressure systems are expected to increase drought severity. The projected 21^st^ century growth changes across Europe indicate serious ecological and economic consequences that require immediate forest adaptation.

## Abstract (web summary)

Tree ring data from a network of beech tree stands across Europe show evidence for a recent growth decline from 1986-2016 and project up to 50% growth reductions in some areas of Europe with future climate change.

## Introduction

Global environmental change is affecting ecosystems in many regions around the world. Forests are key terrestrial ecosystems where evidence increasingly points towards cascading impacts related to anthropogenic-induced climate change^1–3^, including far-reaching consequences for the water and carbon (C) cycles, and services to society^4^. Evolving questions related with those impacts can be best addressed through large-scale analyses, encompassing the full distribution range of key species^3^.

There is a long tradition of forest cover prediction research focus on understanding the links between climate change and forest presence/abundance^5,6^. Less knowledge is available on ecologically-based predictions of species growth performance. Considering that the stem represents ~70% of the tree’s biomass^7^, secondary growth can be considered a reasonable proxy of total C sequestration^7^, and can be effectively used as an indicator of tree health and performance^8^.

Dendroecological analyses typically present local data and have provided valuable regional insight into growth responses to local habitat conditions and climate change^2,9^. Despite recent advances in tree-ring research^9^, spatio-temporal studies of actual and predicted growth are uncommon, particularly at scales incorporating species’ geographic and climatic distributions^10^. The tree-ring community has developed international dendrochronological databanks, yet these are typically biased or limited for certain taxa, biomes and trailing-edge populations^11–13^, hindering their value for ecologically-focused application. If such challenges can be overcome, the large spatial scale represented by tree-ring networks, their annual resolution and the potential for multi-decade assessment of growth changes present a unique opportunity to unravel spatial patterns and drivers of recent growth, and predict future growth dynamics based on climate change scenarios. Such information would be crucial to estimate species resilience to warmer and potentially drier future conditions.

**Fig. 1:**
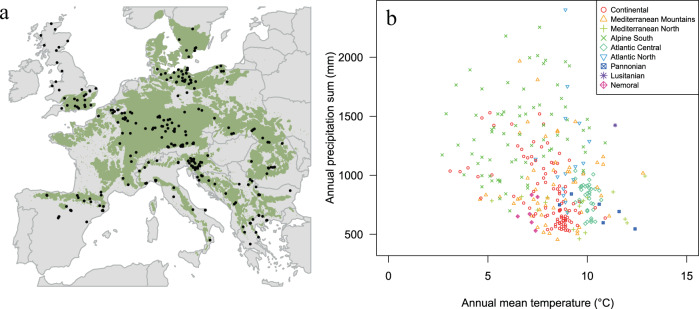
Spatial and climatic range of beech sites. **a** Geographical distribution of the 324 study sites (black dots) in the natural distribution range of European beech (green area based on the EUFORGEN map^65^; see Supplementary Data 2 for details). **b** Climatic envelope of European beech sampling sites, considering annual temperature and precipitation. Sites are labelled according to the environmental zones detailed in Metzger et al.^69^.

Successful upscaling of tree-ring data, however, requires dense networks covering the full range of bioclimatic and ecological conditions of the study species. To enable this advance, we established a dense and species-specific network supportive of comparative ecological analyses, covering the entire ecological spectrum of *Fagus sylvatica* L. (hereafter beech), including over 780,000 ring width measurements from 5800 trees and 324 sampling sites across Europe (Fig. 1).

Beech is one of the most important forest species in Europe from an ecological (e.g. habitat, biodiversity) and socio-economical (e.g. timber harvest, recreation) perspective^14^. Beech played a dominant role in postglacial reforestation, rapidly spreading from its Mediterranean refuges to the central and northern regions of the continent^15^. Currently, in the face of rapid climate change, beech may be endangered in its geographical and ecological range^16^. However, the species’ resilience to predicted changes and its ecological plasticity across the distribution range are not well understood.

Using this network, we analyse past growth rates of beech and use this information to project growth variability considering different climate change scenarios (i.e, representative Shared Socioeconomic Pathways scenarios of CMIP6 (Coupled Model Intercomparison Project)) to disentangle 21st century patterns of the species’ performance at continental scales. We perform a comparative analysis among regions in Europe and map forest growth considering local environmental stresses and disturbances. A generalised linear mixed-effects model (GLMM) is developed to model tree growth and support spatio-temporal comparisons across the species’ distribution range, identifying regions where growth has increased or declined in recent decades. The model is validated and used to predict radial growth during three distinct periods until the late 21st century and the results discussed considering likely climate change scenarios.

Our study provides evidences of striking changes in growth patterns of this species during the studied period, especially in the southern areas. The models showed that growth is being recently limited due to climate and modulated by site-prevailing conditions. In this sense, forecasted reductions in precipitation and temperature increments would lead to an overall decrease in tree productivity, most notably if both conditions occur at the same time. Interestingly, tree growth could be significantly enhanced at high latitudes in the future, even under a worst-case climate change scenario.

## Results

### Model development and performance

Among the tested mixed-effects models, the full model containing all considered and significant variables and interactions was the most accurate to predict the species’ growth across Europe, as shown by the Akaike Information Criterion (AIC) scores (Table 1). AIC measures the relative goodness of fit of a given model; the lower its value, the more accurate the model is.. Indeed, 86% of growth variability was explained by the model (Supplementary Fig. 1). We modelled annual basal area increment (BAI) for 324 beech sites across Europe considering (i) prevailing moisture/aridity conditions, (ii) elevation and latitude to estimate radiation and photoperiod, and (iii) seasonally varying climate conditions including precipitation totals and temperatures from previous-year summer to current-year autmn (relative to the year of tree-ring formation). The GLMM included a total of 21 variables organised in three, interacting variable groups. This resulted in a total of 66 variable interactions that significantly contributed to the growth model (Supplementary Data 1). When fitting the GLMM, estimated previous-year BAI was considered as random factors to account for size dependency of growth trends.

**Table 1:** Models’ validation.

	AI	Cli	Geo	RE	AIC	∆AIC	Chisq	Df	Pr
Null model	–	–	–	●	642591.5	25404.4	NA	NA	NA
	●	–	–	●	642509.6	25322.5	83.9	1	5.32E-20
	–	–	●	●	642417.9	25230.8	95.8	2	1.61E-21
	●	–	●	●	642348.3	25161.2	77.6	4	5.66E-16
	–	●	–	●	624421.0	7233.9	17949	11	0
	●	●	–	●	621819.4	4632.3	2639.6	19	0
	–	●	●	●	618820.9	1633.9	3038.4	20	0
Full model	●	●	●	●	617187.1	0.0	1647.9	7	0

Each row represents a single model and each colored column represents the inclusion of each group of predictor variables (red, moisture conditions (AI), blue, seasonal climatic temperature and precipitation variables (Cli), yellow, geographical variables (Geo) and green, random effects (RE)). For each model, Akaike Information Criterion value and difference (AIC, ∆AIC), *χ*^2^ test value and degrees of freedom (Df) of the *χ*^2^ test and p (Chisq, Pr > Chisq)) are shown.

Application of the GLMM demonstrated that the interaction of geographical variables as latitud or altitud with the aridity index (AI) were significant to explain beech growth variability (i.e. the effects of e or latitude were different between trees growing in xeric and mesic climates). Precipitation correlated positively with tree growth, whereas maximum and minimum temperatures showed variable effects and depended on the season. Seasonal temperatures effects were stronger in explaining growth variability across the distribution range than precipitation.

Contributions of variable interactions to model beech growth were relevant. Regardless of the specific weight and significance of each seasonal climatic variable, our results show that most sensitivities to annually varying climate are modulated by mean aridity and the geographic components altitude and latitude. The final model was able to reproduce tree-ring growth across Europe and covering the entire species distribution for every year for the period 1955–2016 (Supplementary Fig. 1).

### Past regional growth changes

**Fig. 2:**
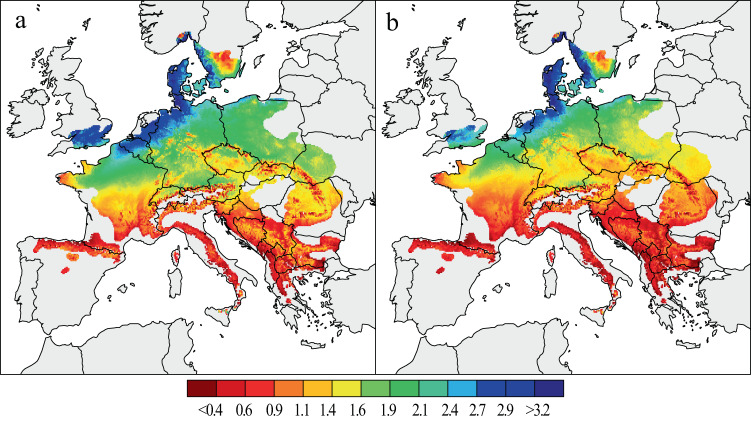
Spatial patterns of beech growth during the last decades. Mean estimates of BAI (in mm^2^) from 1955–1985 (**a**) and 1986–2016 (**b**), calculated for a theoretical tree derived from a 324-site chronology network.

To compare beech forest performance over past decades, mean growth rates of two consecutive 31-year periods were computed for a population-wide average beech tree with a fixed basal area of 86059.03 (1/10,000 mm^2^) (i.e. the average basal area of the entire data set, which is equivalent to a 80 years old tree) (Fig. 2). This multidecadal aggregation was chosen as it represents an unprecedented increase in temperatures from 1955–1985 to 1986–2016 exceeding 1 °C in many regions in Europe^17^. Indeed, the most recent period is the warmest 31-year period in Europe over the past 500 years, and is up to 0.45 °C warmer than the second warmest 31-year period, which occurred from 1750 to 1779^18^.

**Fig. 3:**
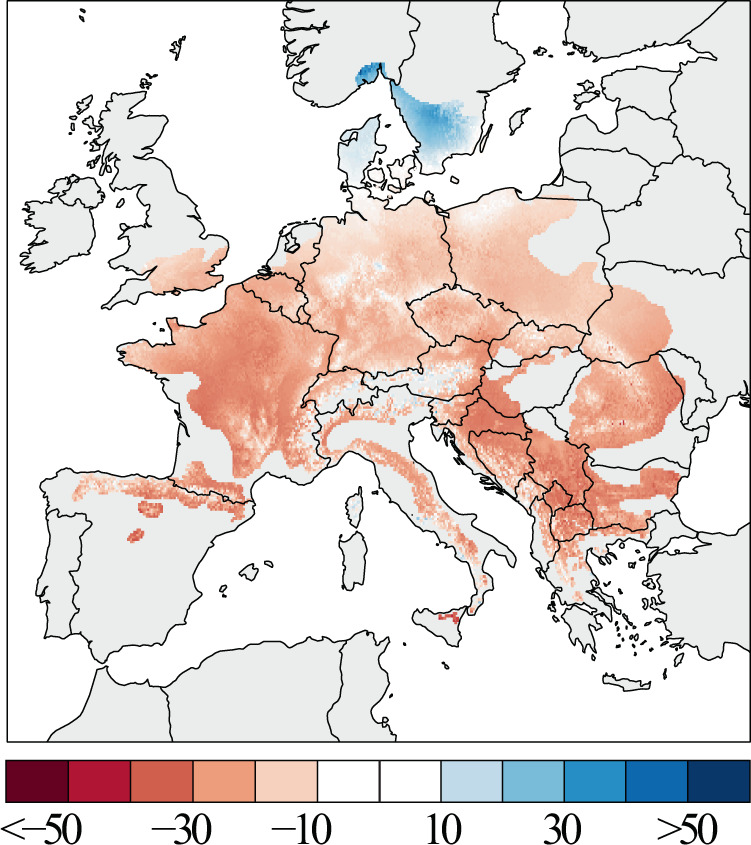
The spatial pattern of beech growth changes across Europe. Tree growth changes are expressed in percent BAI change from 1986 to 2016 relative to the 1955–1985 period mean.

Our results reveal substantial spatio-temporal differences in beech growth over the past six decades across Europe (Fig. 3). Tree growth rate was two to three times higher at low altitudes in NW and central Europe including coastal sites in Belgium, Netherlands, Denmark and the British Isles, compared in the southern distribution limit of beech. Lower tree growth is also modelled at higher altitudes in central Europe, the Alps, and along the Carpathians. Growth was lowest at the northernmost and south-western edge of the species’ distribution in Sweden and Spain, respectively, as well as in Italy and south-eastern Europe. The most recent period showed a similar geographical pattern in tree growth, compared to 1955–1985. However, regions of high growth are overall less extensive and regions of reduced growth overall larger, and these tendencies are superimposed on a general decrease in growth magnitude.

The spatial representation of growth differences between 1955-1985 and 1986–2016 reveals a notable decline in growth across most of the area covered by European beech (Fig. 3). The strength of this decline varies across Europe, being higher at low latitudes and lower towards north, thereby revealing a distinct latitudinal gradient of forest growth decline. The sharpest contrast was recorded between northern areas including Sweden and Norway, where modelled growth increase up to 20% between the two periods, and south Europe, where severe growth declines of up to −20% were modelled.

### 21st century growth responses to climate change scenarios

**Fig. 4:**
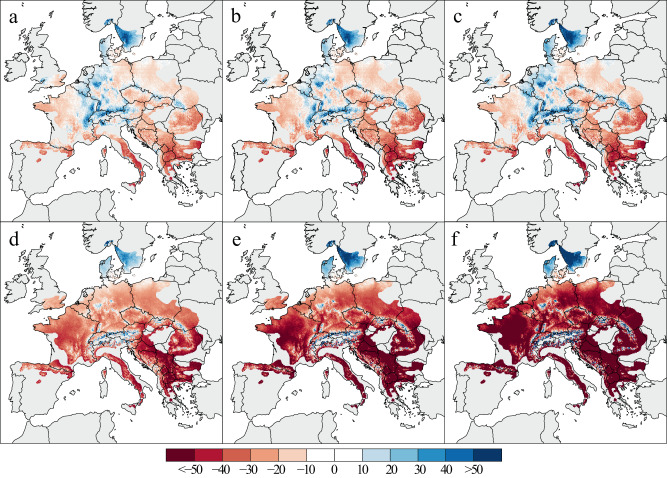
Relative changes in tree growth. Changes are projected under SSP1-2.6 (**a**–**c**) and SSP5-8.5 (**d**–**f**) CMIP6 climate scenarios for different periods: 2020–2050 (**a**, **d**), 2040–2070 (**b**, **e**), and 2060–2090 (**c**, **f**) In this panel, BAI changes were expressed in percentage of change compared to the 1986–2016 period.

The GLMM yields varying BAI trends under the projected climate change scenarios (Fig. 4). Even under the relatively optimistic SSP1-2.6 scenario, growth changes across geographic gradients remain greater in magnitude than the observed changes in growth between the two historical periods. Growth reductions up to 30% are projected in southern Europe during the 2020-250, compared to a baseline of 1986–2016 (Fig. 4a). This decline increases northward to reductions of ~10% and then zero change prevailing in central European sites. On the other hand, growth increases of ~25% are projected in mountainous environments across central Europe, and ~35% increases are expected for southern Scandinavia. Patterns from 2040–2070 and 2060–2090 (Fig. 4b, c) are similar, except for more accentuated growth reductions in southern Europe including the Balkans, and more polarised patterns (e.g. in the Apennines, Greece, Romania and Spain *versus* the Alps, Sweden and Denmark) towards the end of the 21st century.

The more realistic SSP5-8.5 scenario leads to dramatic decreases in beech productivity over vast parts of Europe (Fig. 4d–f). From 2020 to 2050 growth, decreases of 20–30% are expected to affects most forests in central Europe, even including some elevated sites in northeast France and southern Germany. In southern Europe, growth reductions may exceed 50%, particularly during the period 2040–2070. On the other hand, north of 55°N and in mountainous regions of Central Europe, growth trends are positive. These spatially varying trends continue throughout the 2040–2070 and 2060–2090 periods, though at much accelerated rates. From 2040 to 2070, the general southeast-to-northwest pattern, modulated by altitude, is pronounced, including maximum growth reductions >50% in southern Europe. The dramatic changes modelled from 2060 to 2090 considering SSP5-8.5 should be interpreted with caution, as the altered climatic conditions in some regions exceed the applicability domain of the GLMM (Supplementary Fig. 2).

## Discussion

Our study provides evidence of striking changes in growth patterns of a European key tree species over the past 60 years and upcoming 80 years. Over recent decades, growth declines are particularly severe towards the southern distribution limits in Europe, and these general trends will continue as the climate continues to warm and become drier. GLMM models demonstrate how spatial differences in growth are predominantly explained by differences in temperature and water availability, all modulated by site-prevailing climate conditions. Consequently, reductions in precipitation or increases in temperature will lead to an overall decrease in tree productivity, most notably if both conditions occur simultaneously. Importantly, our results help to reconcile previous results, which had failed to provide a consistent picture of growth trends in beech across Europe, particularly in southern Europe where predicted declines were not consistently reported in site-based analyses. Here we show that when age/size effects are accounted for, growth declines in beech are observed across southern Europe, particularly at lower elevation. Furthermore, our results generalise recent reports of growth declines extending into central Europe. In this sense, our study reconciles differences across studies and provides a comprehensive approach revealing a persistent decrease in beech tree productivity and C sequestration since the 1980s in all but the most northern of the species distribution.

Adaptive management is of major importance for preserving forest viability and mitigating harmful effects of climate change. To invoke such management policies, we need to assess species-specific climatic effects at varying spatio-temporal scales^19,20^. In this sense, empirical modelling has proven useful to forest managers to anticipate climate impacts on future forest growth, supporting, for example, species selection in case of tree plantations, or planning assisted migrations^21,22^. Therefore, dendroecology combined with modelling is a powerful tool to evaluate the environmental imprints on mid to long-term forest dynamics, and opens the possibility to estimate tree productivity, and associated functioning under projected climate and site conditions^10,23^. The GLMM model applied here also addresses possible sampling biases that commonly apply in field-based applications (e.g., the big-tree selection bias^24^), as it takes into account and minimise the effects of size and age in statistical analysis by adding tree size as random factor. Potential biases can occur, however, if sample sizes are small and if age cohorts were equal across sites^24^.

The climatic and geographical variables included in models for continent-scale growth predictions must be ecologically meaningful and accessible. Although other variables affect growth variability of beech, such as soil type, nutrient presence, masting events, competition, and insect infestation^25–31^, most of these are difficult to predict. Evaluating their impact on growth is also challenging in spatial modelling, often because of limited spatial resolution, and may depend directly or be correlated with other variables. For example, photoperiod determines the seasonality of processes in trees, the length of xylogenesis, and therefore the amount of growth^32^, yet it is closely correlated with latitude. The interaction among variables must be considered given the variability of climate sensitivity of a species across environmental, altitudinal and latitudinal gradients^33–36^.

The GLMM growth model applied here displays strong geographical variance and reveals the existence of a regional optimum for beech growth in mountainous areas of central Europe, under the current climate. The spatial variability of beech growth across Europe follows an apparent combination of N-S and NW-SE gradients, combined with an altitudinal gradient. In this sense, beech is more productive (i.e. produce wider rings) at lower elevations, particularly in NW Europe. Indeed, beech phenology, as well as rates and timing of xylogenesis, are affected by altitude^37–39^, which in turn control tree growth. The evaluation of the impact of the warmer and drier conditions over recent decades across the species’ distribution requires consideration of local differences from regional climate and site conditions. The observed N-S and NW-SE growth gradients across Europe may be affected by prevailing atmospheric circulation patterns, continentality and photoperiod optimum, but further research is needed to disentangle the drivers of growth variability across these large scales, geographic gradients.

Subsequent to a tree growth increase during the first part of the last century in Europe^40^, recent studies reported growth decreases in beech^41,42^. This decrease was attributed to increasing temperatures, the impact of extreme climatic events and long-term changes of environmental conditions. Our findings of negative beech BAI trends over past decades are inconsistent with other studies reporting growth increases^31,43,44^ and spatially varying growth trends depending on altitude^45^. However, the different findings are mainly due to varying approaches when dealing with age effects, and whether the results are derived from repeated diameter measurements and detrended chronologies instead of raw tree growth increments. Despite methodological differences, local case studies are relevant as they may account local trends, which could help to identify research gaps and further research^46^. In this sense, our study reconciles differences across studies and provides a comprehensive approach revealing a persistent decrease in beech tree productivity and C sequestration since the 1980s. Although beech has been reported to be drought sensitive throughout Europe^34,42,47^, our simulations suggest that temperatures may start to gain prominence as a limiting factor across a large portion of the species’ distribution area. Our results support those of Mette et al. (2013)^48^ showing that beech growth in central Europe is currently not only limited by precipitation. The observed and projected temperature increases foster atmospheric pressure deficits, constrain stomata closure, amplify tree water demand and increase risks of hydraulic failure^41^. Drought induced defoliation, extension of canopy duration and associated limitations of metabolic reserves (where respiration may exceed photosynthesis^49^), and higher turnover rates of fine roots likely contribute to this temperature sensitivity^50^. Thus, even though beech is a late-successional species that is considered competitively superior to many other European tree species^51^, including broad-leaved *Quercus*, *Acer*, *Tilia*, *Fraxinus* and *Carpinus*^42^, it is also prone to warming-induced growth declines.

Our results also demonstrate that the effects of warming temperatures, especially beyond 1.5 °C, cannot be compensated without large increases in precipitation (as in the SSP1-2.6 scenario, see Supplementary Fig. 3), except at very high altitudes in central Europe. Similar conclusions were drawn by Walentowski et al.^20^ demonstrating that temperature rises must be compensated by increases in precipitation to maintain tree vitality. Severe growth reductions are expected if the combination of summer drought and hotter temperatures becomes prevailing, as is forecasted in SSP5-8.5. The cumulative effect of “hotter droughts”^52^ might lead to amplified hydraulic failure and dieback of vast forested areas. The highest vulnerability of beech sites to global warming is observed at the southern edge of the species’ distribution range, as shown by Forzieri et al.^3^.

The projected increase of global mean surface temperature by the end of the 21st century is expected to range from 1.5 to 5.5 °C (with respect to 1900–2000) depending on the Shared Socioeconomic Pathways scenario. The projected changes in precipitation will not be uniform, but include decreases in southern Europe and increases in northern latitudes (north of 55°N)^1^. However, future climate is uncertain, particularly for precipitation, as the CMIP6 archive might be subject to multiple sources of error. Our results are likely affected by additional uncertainties including the role of extreme weather events (i.e. late spring frost, heat waves, fires), soil composition (i.e. nitrogen, phosphorous, potassium) and tree species competition, all of which complicating species-specific tree growth projections.

Projected climate change will foster a progressive decrease of beech growth. As beech is a dominant tree species across large regions of Europe’s forests, this indicates an important reduction in functioning as a carbon sink to mitigate atmospheric CO_2_ increases. Furthermore, as beech is high importance both commercially and environmentally, a long-lasting decrease in productivity may be critical at multiple levels. We recommend to forest managers to consider these results in long-term silviculture plannings.

## Conclusions

Analysing the drivers of growth across an unprecedented network of beech sites covering Europe, we report a pervasive growth rate decline from 1955 to 2016. This decline is widespread in Europe, except for sites located towards the northern distribution range in Denmark, Norway and Sweden and at higher elevation in mountain regions. Recorded growth variations range from +10–20% in the north, to −20% in the south of Europe. By employing a GLMM, we show that future increases in global temperature, particularly those exceeding 1.5 °C, lead to a widespread decrease −20 to −40% in beech growth, a situation that could be further amplified to −50% if drought conditions prevail. These significant growth trends point towards increased forest mortality, as declining growth has been reported as a precursor of tree die-off^2^. These findings challenge recent predictions of increasing terrestrial carbon stocks under climate change scenarios^53^, as the strength of beech forests as a carbon sink will decrease.

## Methods

### Tree-ring network

We compiled a network of tree-ring chronologies from closed-canopy and mature stands dominated by European beech. The databank comprises 324 sites, with ~5800 trees and ~780,000 tree-ring measurements. Geographically, the networks extends from 5.8 to 28.4°E and 38.8 to 58.5°N, and covers the entire geographic distribution range of *Fagus sylvatica* in Europe. Sites also cover the full climatic range of the species, with annual precipitation and temperature ranging from 500 to 2000 mm and 3.8 to 13.5 °C, respectively (Fig. 1 and Supplementary Data 2). The selected plots are mostly undisturbed sites, located between 1 and 1900 m a.s.l, covering the full elevation gradient of the species, including beech treeline sites.

Increment cores were dried and sanded according to standard procedures^54^ to enhance the visibility of tree-ring boundaries. Tree-ring widths were measured to the nearest 0.01 mm and each tree-ring series were crossdated using COFECHA or CooRecorder software. Classical detrending methods to remove age-related trends were not applied to support comparisons between different periods. We instead converted the tree-ring width data into annual basal area increments (BAI), in cm^2^ per year, as this procedure accounts for the geometrical constraint of adding a cross-sectional area of wood to a stem of an increasing radius. The BAI series of each tree were obtained based on the measured diameter at breast height when sampled, and computed using the bai.out function of the R package *dplR* (version 1.7.2). The mean BAI of defined periods can be compared over time, as it is not affected by biological trends^55,56^.

### Climate variables

CHELSAcruts^57^ was used to extract climate data from gridded networks. Monthly precipitation and maximum and minimum temperatures were downloaded and combined to seasonal means covering the period from 1901 to 2016. Prevailing moisture conditions were defined by applying the De Martonne aridity index^58^ (AI) as previous studies showed that site-specific moisture conditions modulate the climate sensitivity of trees^59^. AI was calculated for European grid cells from 1950 to 2016 using (Eq. 1):

$$AI\,=\,\frac{P}{10+T}$$

where *P* is the annual precipitation sum (in mm) and *T* (in °C) the annual mean air temperature. The climate types defined by De Martonne range from arid (values from 0 to 10), semi-arid (10–20), Mediterranean (20–24), semi-humid (24–28), humid (28–35), very humid (35–55) to extremely humid (>55).

### Predictive growth model

Generalised linear mixed-effects models (GLMM) were used to estimate the joint effects of climate and geographical variables on tree growth. In the statistical computing environment R, GLMMs were fitted by maximum likelihood (Adaptive Gauss-Hermite Quadrature) using the R package *lme4* (version 1.1–21). These models are particularly useful as they combine the properties of linear mixed models and generalised linear models, allowing the inclusion of random effects and the analysis of nonnormal data^60,61^. Mixed models are suited for studies over time influenced both by factors that can be assumed to be similar for many sites (e.g. the effect of climate) and by characteristics that substantially vary from site to site (e.g. populations)^62^. In addition, mixed models explicitly account for the correlations between repeated measurements within each site. In fact, collinearity among predictor variables can cause problems in model’s variables interpretation because those predictors explain some of the same variance in the response variable, and their effects cannot be estimated independently^63^. Since the main objective of model application is the interpretation of the output (i.e. growth models), nor the influence of the variables, we included variables based on AIC values (Table 1).

The model was based on the period 1950–2016 due to the common availability of climate and dendrochronological data. We then fitted a single GLMM to predict annual BAI of a tree *j* in a site *i* in a year *t* as a function of prevailing climate, latitude, altitude, temperature and precipitation (season *k*), assuming a gamma distribution of the response variable (Eq. 2):

$$\log ({{{{{\mathrm{BA}}}}}}{{{{{{\mathrm{I}}}}}}}_{i,j,t})\,= 	\,\,\beta +\,\log ({{{{{\mathrm{A}}}}}}{{{{{{\mathrm{I}}}}}}}_{i})+f({{{{{\mathrm{LA}}}}}}{{{{{{\mathrm{T}}}}}}}_{i})+f({{{{{\mathrm{AL}}}}}}{{{{{{\mathrm{T}}}}}}}_{i})+f(T{{{{{\mathrm{ma}}}}}}{{{{{{\mathrm{x}}}}}}}_{i,t,k})+f(T{{{{{\mathrm{mi}}}}}}{{{{{{\mathrm{n}}}}}}}_{i,t,k})+\,\log ({{{{{\mathrm{P}}}}}}{{{{{{\mathrm{P}}}}}}}_{i,t,k})\,\\ 	+\,\log ({{{{{\mathrm{A}}}}}}{{{{{{\mathrm{I}}}}}}}_{i})\times f({{{{{\mathrm{LA}}}}}}{{{{{{\mathrm{T}}}}}}}_{i})+\,\log ({{{{{\mathrm{A}}}}}}{{{{{{\mathrm{I}}}}}}}_{i})\times f({{{{{\mathrm{AL}}}}}}{{{{{{\mathrm{T}}}}}}}_{i})+f({{{{{\mathrm{LA}}}}}}{{{{{{\mathrm{T}}}}}}}_{i})\times f({{{{{\mathrm{AL}}}}}}{{{{{{\mathrm{T}}}}}}}_{i})+\,\log ({{{{{\mathrm{A}}}}}}{{{{{{\mathrm{I}}}}}}}_{i}) \\ 	\times \,f(T{{{{{\mathrm{ma}}}}}}{{{{{{\mathrm{x}}}}}}}_{i,t,k}),f(T{{{{{\mathrm{mi}}}}}}{{{{{{\mathrm{n}}}}}}}_{i,t,k}),\,\log ({{{{{\mathrm{P}}}}}}{{{{{{\mathrm{P}}}}}}}_{i,t,k})+f({{{{{\mathrm{LA}}}}}}{{{{{{\mathrm{T}}}}}}}_{i})\times f(T{{{{{\mathrm{ma}}}}}}{{{{{{\mathrm{x}}}}}}}_{i,t,k}),f(T{{{{{\mathrm{mi}}}}}}{{{{{{\mathrm{n}}}}}}}_{i,t,k}),\,\log ({{{{{\mathrm{P}}}}}}{{{{{{\mathrm{P}}}}}}}_{i,t,k}) \\ 	 +\,f({{{{{\mathrm{AL}}}}}}{{{{{{\mathrm{T}}}}}}}_{i})\times f(T{{{{{\mathrm{ma}}}}}}{{{{{{\mathrm{x}}}}}}}_{i,t,k}),f(T{{{{{\mathrm{mi}}}}}}{{{{{{\mathrm{n}}}}}}}_{i,t,k}),\,\log ({{{{{\mathrm{P}}}}}}{{{{{{\mathrm{P}}}}}}}_{i,t,k})\,+({{{{{\mathrm{B}}}}}}{{{{{{\mathrm{A}}}}}}}_{j,i,t}|{{{{{\mathrm{Cod}}}}}}{{{{{{\mathrm{e}}}}}}}_{j})$$

Where *β* is the intercept, *f* are smoothing functions and log are logarithms applied to the variables. All variables were standardised before model constructions to guarantee a compensated weight and avoid effects related with the range of variables. The elements included in the model as independent variables were AI (Aridity Index), LAT (latitude), ALT (altitude), Tmax (maximum seasonal temperatures from previous to current summer), Tmin (minimum seasonal temperatures from previous to current summer), and PP (total seasonal precipitation from previous to current summer), as well as the statistically significant interaction between variables. To account for the possible influence of age effects (i.e. trends), and particularities of individual trees, we additionally included a random slope of previous year basal area (BA) of each ring and tree (Code). Therefore, BA and tree code were included as random factors in the model to avoid individual influences on our results. The model was evaluated considering the dominant paradigm of GLMM validation^64^, which involves the generation of a null hypothesis (i.e. null model) to test the selected model through a chi-squared test (*P* < 0.05). We thereby evaluated the accuracy of the model (full model) using a likelihood ratio test by comparing the model with reduced models where explanatory variables of interest were omitted, before finally a comparison with a “null” model was performed, where only the intercept term and random effects were included (Table 1).

Later, the model was applied to each cell of a climatic grid covering the entire species range, based on EUFORGEN distribution maps^65^. Annual BAI values from 1950 to 2016 were calculated to compare mean growth rates over Europe, i.e. mean BAI values of the periods from 1955 to 1985 and 1986 to 2016. The turning point in the mid-1980s was selected as this represents the onset of an ongoing period of strong warming^17,18^. Percentage growth changes^31^ were calculated for each grid point by comparing mean growth rates with pre-defined periods. All maps were produced using R package *maps* (version 3.3.0).

### Simulated growth considering climate change scenarios

We used CMIP6 multi-model ensemble means representative of various earth system models for minimum and maximum temperature (21 models) as well as precipitation (26 models) to project future tree growth, representative of an optimistic (SSP1-2.6) and a pessimistic (SSP5-8.5) scenario^66^. To do so, we for each scenario-model combination computed the difference of variable-specific climatologies between historic simulations (period 1985–2014) and future simulations representative of three distinct periods (2020–2050, 2040–2070, 2060–2090) and averaged those over all models for each scenario to obtain ensemble means. These ensemble mean delta-values were then added to the corresponding CHELSAcruts^67^ climate variables, to obtain climate data representing simulated climatologies of the corresponding scenario and period.. Therefore, all seasonal climatic variables of the model were updated to future projected predictions (depending on the SSP), meanwhile geographic variables and AI index remained stable. Future beech growth was forecasted by applying the model to projected local climatic conditions, resulting in six growth variation scenarios.

Given the range of the climate scenarios, we calculated applicability domains (AD)^59,68^ of the model for each period (Supplementary Fig. 2). When the range of future climate variability exceeded the range of past conditions from 1901 to 2016, the predictive performance of the model becomes uncertain. Therefore, for different combinations of seasonal climate conditions located within the AD, growth estimates are expected to be as reliable as those in the training sample. However, for those pixels outside the AD, the reliability of estimates declines, and the predicted growth patterns should be interpreted with caution.

### Reporting summary

Further information on research design is available in the Nature Research Reporting Summary linked to this article.

## Supplementary information

Supplementary Information

Description of Additional Supplementary Files

Supplementary Data 1

Supplementary Data 2

Supplementary Data 3

Supplementary Data 4

Supplementary Data 5

Reporting Summary

## Data Availability

The data that support the findings of this study are available from the corresponding author and co-authors upon reasonable request. All relevant data for the figures are included in the supplementary information files.
