# Supplementary material for: Climate-change-driven growth decline of European beech forests
Source: Commun Biol. 2022 Mar 10;5:163. doi: 10.1038/s42003-022-03107-3 (PMC8913685; doi:10.1038/s42003-022-03107-3)

Supplementary Figure 1. Smooth scatter plot of observed by predicted BAI values using the growth model.

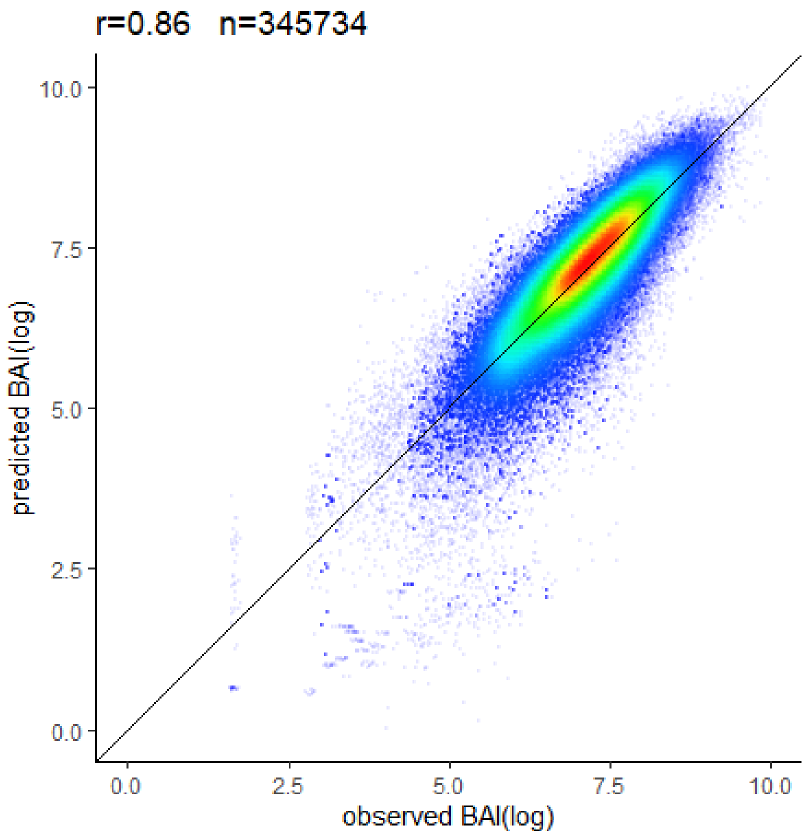

Supplementary Figure 2. Geographical representation of the applicability domains corresponding to different periods and climate change scenarios. Pixels within and outside the applicability domain are shown in green and grey, respectively. a) SSP 1-2.6 from 2020-2050, b) SSP 1-2.6 from 2040-2070, c) SSP 1-2.6 from 2060-2090, d) SSP 5-8.5 from 2020-2050, e) SSP 5-8.5 from 2040-2070, and f) SSP 5-8.5 from 2060-2090.

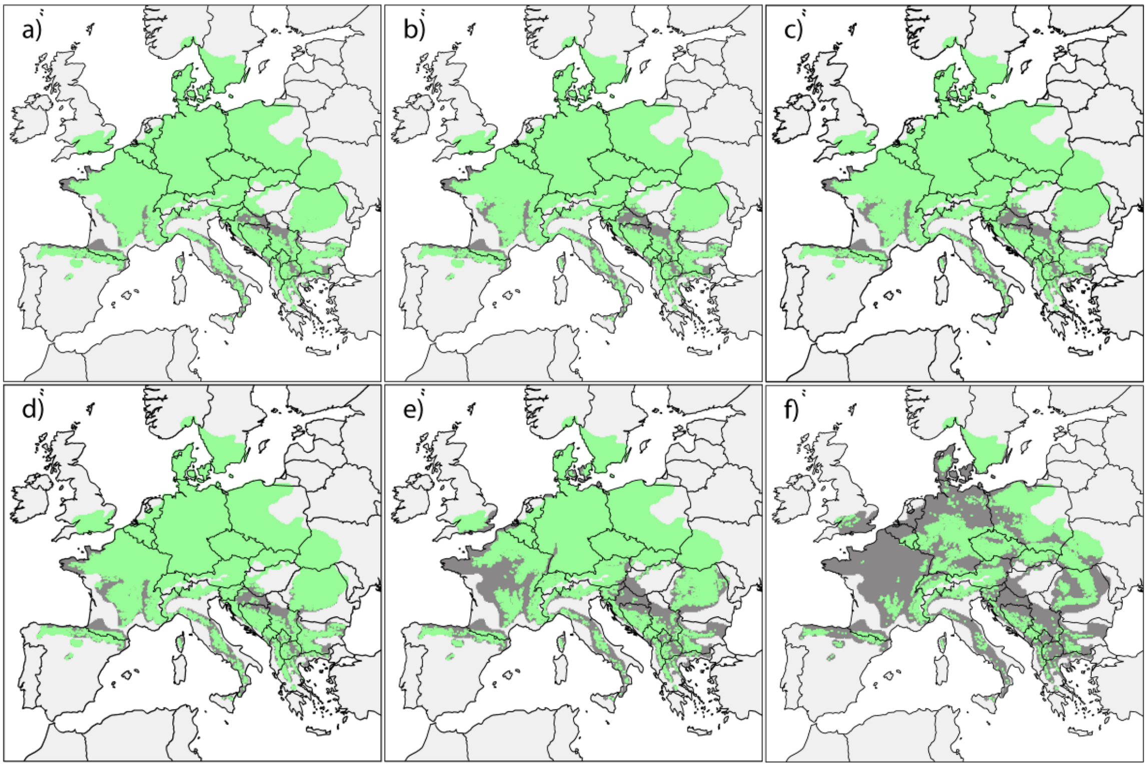

Supplementary Figure 3. Delta values for precipitation (PCP) and maximum (Tmax) and minimum temperatures (Tmin) of projected climate change scenarios by seasons. The delta values of the SSP1-2.6 scenario (blue) and those for the SSP5-8.5 (red) are grouped by seasons and divided by the predefined periods. The central lines of boxplots indicate the median value, vertical hinges indicate first and third quartiles, error bars indicate the 95% confidence interval of the median and dots indicate outliers (i.e. values beyond the 95% confidence interval threshold).

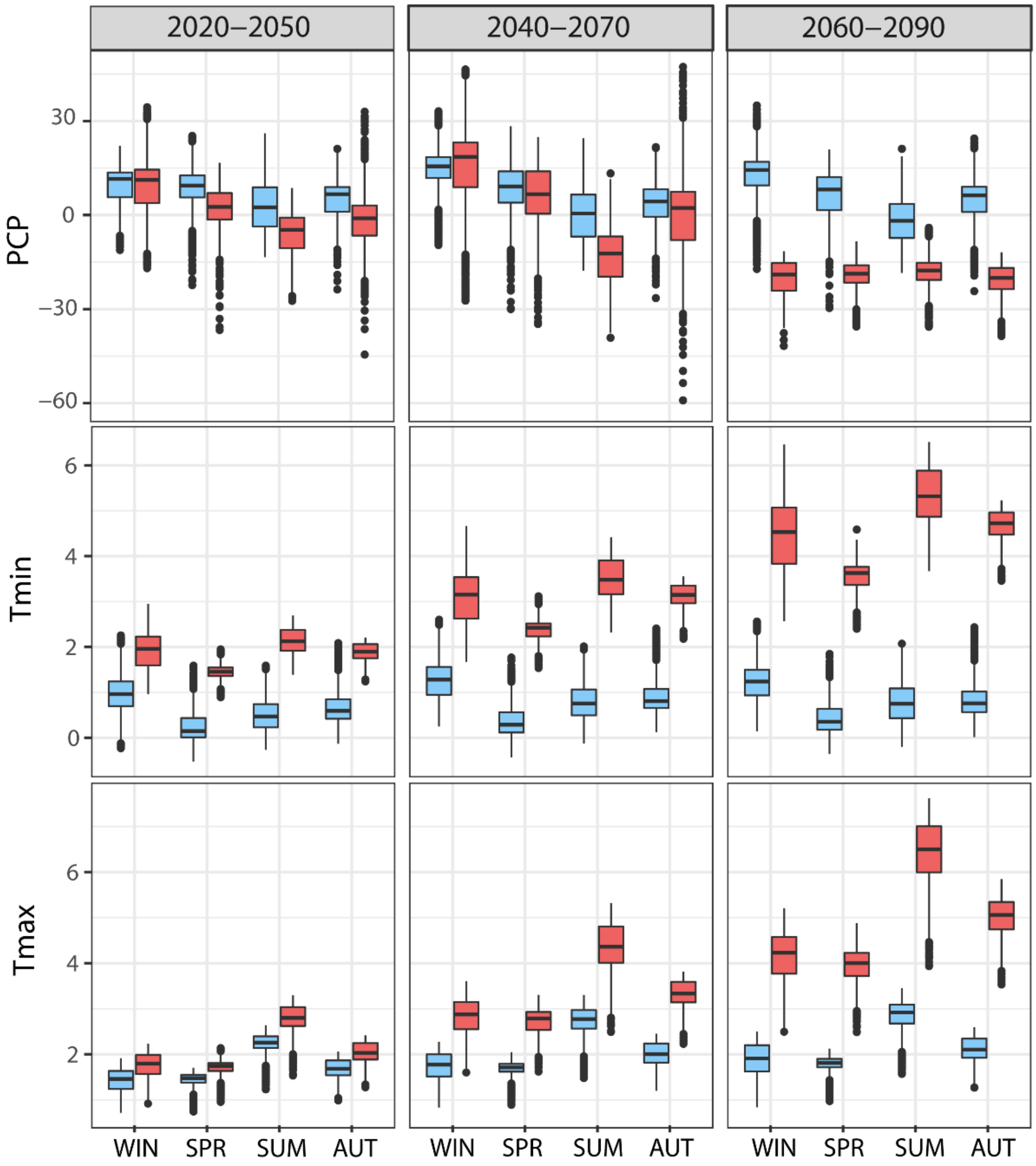

Supplement: Supplementary file 1 — Supplementary Information [file 42003_2022_3107_MOESM1_ESM.pdf]
