# Supplementary material for: Climate-change-driven growth decline of European beech forests
Source: Commun Biol. 2022 Mar 10;5:163. doi: 10.1038/s42003-022-03107-3 (PMC8913685; doi:10.1038/s42003-022-03107-3)
Supplement: Supplementary file 2 — Description of Additional Supplementary Files [file 42003_2022_3107_MOESM2_ESM.pdf]

# Description of Additional Supplementary Files

**File name:** Supplementary Data 1
**Description:** Model fixed effects of variables. *** is p = 0- 0.001, ** p = 0.001-0.01, ** p = 0.01-0.05*, '.' p = 0.05-0.1. LAT is latitude, ALT altitude, AI De'Martonne Aridity Index, T temperature, PCP precipitation, MAX mean maximum temperature, MIN mean minimum temperature, p previous, SPR is spring, SUM summer, AUT autumn and WIN winter.

**File name:** Supplementary Data 2
**Description:** Beech network site characteristics. A code (ID), coordinates (Lat, Lon), altitude (Alt) and last measured ring is provided for each sampled site.

**File name:** Supplementary Data 3
**Description:** Source data for Figures 1 and 2.

**File name:** Supplementary Data 4
**Description:** Source data for Figure 3.

**File name:** Supplementary Data 5
**Description:** Source data for Figure 4.
