# Supplementary material for: Climate-change-driven growth decline of European beech forests
Source: Commun Biol. 2022 Mar 10;5:163. doi: 10.1038/s42003-022-03107-3 (PMC8913685; doi:10.1038/s42003-022-03107-3)
Supplement: Supplementary file 8 — Reporting Summary [file 42003_2022_3107_MOESM8_ESM.pdf]

nature research

Corresponding author(s): Edurne Martinez del Castillo

Last updated by author(s): Jan 5, 2022

# Reporting Summary

Nature Research wishes to improve the reproducibility of the work that we publish. This form provides structure for consistency and transparency in reporting. For further information on Nature Research policies, see our Editorial Policies and the Editorial Policy Checklist.

## Statistics

For all statistical analyses, confirm that the following items are present in the figure legend, table legend, main text, or Methods section.

| n/a | Confirmed | |
|---|---|---|
| ☐ | ☒ | The exact sample size ($n$) for each experimental group/condition, given as a discrete number and unit of measurement |
| ☐ | ☒ | A statement on whether measurements were taken from distinct samples or whether the same sample was measured repeatedly |
| ☐ | ☒ | The statistical test(s) used AND whether they are one- or two-sided<br>*Only common tests should be described solely by name; describe more complex techniques in the Methods section.* |
| ☐ | ☒ | A description of all covariates tested |
| ☐ | ☒ | A description of any assumptions or corrections, such as tests of normality and adjustment for multiple comparisons |
| ☒ | ☐ | A full description of the statistical parameters including central tendency (e.g. means) or other basic estimates (e.g. regression coefficient) AND variation (e.g. standard deviation) or associated estimates of uncertainty (e.g. confidence intervals) |
| ☐ | ☒ | For null hypothesis testing, the test statistic (e.g. $F$, $t$, $r$) with confidence intervals, effect sizes, degrees of freedom and $P$ value noted<br>*Give P values as exact values whenever suitable.* |
| ☒ | ☐ | For Bayesian analysis, information on the choice of priors and Markov chain Monte Carlo settings |
| ☒ | ☐ | For hierarchical and complex designs, identification of the appropriate level for tests and full reporting of outcomes |
| ☒ | ☐ | Estimates of effect sizes (e.g. Cohen's $d$, Pearson's $r$), indicating how they were calculated |

*Our web collection on statistics for biologists contains articles on many of the points above.*

## Software and code

Policy information about availability of computer code

| | |
|---|---|
| Data collection | TSAP-Win™ from RINNTECH, CooRecorder from Cybis. |
| Data analysis | R-4.1.1 for Windows, Rstudio free |

For manuscripts utilizing custom algorithms or software that are central to the research but not yet described in published literature, software must be made available to editors and reviewers. We strongly encourage code deposition in a community repository (e.g. GitHub). See the Nature Research guidelines for submitting code & software for further information.

## Data

Policy information about availability of data

All manuscripts must include a data availability statement. This statement should provide the following information, where applicable:

- Accession codes, unique identifiers, or web links for publicly available datasets
- A list of figures that have associated raw data
- A description of any restrictions on data availability

The data that support the findings of this study are available from the corresponding author and co-authors upon reasonable request.

# Field-specific reporting

Please select the one below that is the best fit for your research. If you are not sure, read the appropriate sections before making your selection.

☐ Life sciences ☐ Behavioural & social sciences ☒ Ecological, evolutionary & environmental sciences

For a reference copy of the document with all sections, see nature.com/documents/nr-reporting-summary-flat.pdf

# Ecological, evolutionary & environmental sciences study design

All studies must disclose on these points even when the disclosure is negative.

| | |
|---|---|
| Study description | Dendrocronological network covering the entire species range |
| Research sample | Closed-canopy and mature stands dominated by European beech (Fagus sylvatica) |
| Sampling strategy | From one to three increment cores were taken per tree and from 10 to 30 trees were sampled in each site. |
| Data collection | Increment cores were dried and sanded according to standard procedures54 to enhance the visibility of tree-ring boundaries. Tree-ring widths were measured to the nearest 0.01 mm and a calendar year was assigned to each ring. All authors contributed to the data collection. |
| Timing and spatial scale | Data collection is dated from 1993 to 2018 |
| Data exclusions | No data were excluded from the analysis |
| Reproducibility | Results can be reproducible by using a similar dendrochronological dataset and the same climatic data. |
| Randomization | We did not select random trees since the had to be dominant, healthy and representative trees of each sampling site. |
| Blinding | Methods of blinding are not applicable to our study. |

Did the study involve field work? ☒ Yes ☐ No

## Field work, collection and transport

| | |
|---|---|
| Field conditions | The databank comprises 324 sites, with annual precipitation and temperature ranging from 500-2,000 mm and 3.8-13.5ºC, respectively |
| Location | 324 sites across Europe, specified in Fig. 1 and in Supplementary Table 1 |
| Access & import/export | Local and National laws were followed, and the authors had the needed permits to sample in the study sites. Given the size of sampling sites, individual numbers of certificates are not provided. |
| Disturbance | The holes that we made to the trees have a minimum disturbance effects. The holes are usually healed in a year. |

# Reporting for specific materials, systems and methods

We require information from authors about some types of materials, experimental systems and methods used in many studies. Here, indicate whether each material, system or method listed is relevant to your study. If you are not sure if a list item applies to your research, read the appropriate section before selecting a response.

## Materials & experimental systems

| n/a | Involved in the study | |
|---|---|---|
| ☒ | ☐ | Antibodies |
| ☒ | ☐ | Eukaryotic cell lines |
| ☒ | ☐ | Palaeontology and archaeology |
| ☒ | ☐ | Animals and other organisms |
| ☒ | ☐ | Human research participants |
| ☒ | ☐ | Clinical data |
| ☒ | ☐ | Dual use research of concern |

## Methods

| n/a | Involved in the study | |
|---|---|---|
| ☒ | ☐ | ChIP-seq |
| ☒ | ☐ | Flow cytometry |
| ☒ | ☐ | MRI-based neuroimaging |
